# Multicomponent reactions: A simple and efficient route to heterocyclic phosphonates

**DOI:** 10.3762/bjoc.12.121

**Published:** 2016-06-21

**Authors:** Mohammad Haji

**Affiliations:** 1 Chemistry Department, Science and Research Branch, Islamic Azad University, Tehran, Iran

**Keywords:** multicomponent reactions, organophosphorus chemistry, phosphorus reagents, phosphorylated heterocycles

## Abstract

Multicomponent reactions (MCRs) are one of the most important processes for the preparation of highly functionalized organic compounds in modern synthetic chemistry. As shown in this review, they play an important role in organophosphorus chemistry where phosphorus reagents are used as substrates for the synthesis of a wide range of phosphorylated heterocycles. In this article, an overview about multicomponent reactions used for the synthesis of heterocyclic compounds bearing a phosphonate group on the ring is given.

## Introduction

Heterocyclic rings are found in many naturally occurring compounds and they compose the core structures of many biologically active scaffolds as well as some industrial compounds [1–3]. On the other hand, phosphonic acid and its related derivatives are considered as potential bioisosters of the corresponding carboxylic acids [4]. Thus, the incorporation of phosphonyl groups into the heterocyclic systems has led to an important class of organophosphorus compounds that has attracted the attention of both industrial and medicinal chemists [5–12]. Many efforts have been made to prepare these bioactive compounds over the last 60 years [13]. There are two general approaches to the synthesis of heterocyclic phosphonates: (a) the direct electrophilic or nucleophilic phosphorylation of the heterocyclic systems and (b) the ring closure of phosphoryl-functionalized substrates through cyclization or cycloaddition reactions [14–19].

Multicomponent reactions (MCRs) constitute one of the most efficient tools in modern synthetic organic chemistry, since they have all features that contribute to an ideal synthesis: high atom efficiency, quick and simple implementation, time and energy saving, environment-friendly and they offer a target and diversity-oriented synthesis [20]. Therefore, the development of new multicomponent reactions towards biomedical and industrial scaffolds is inevitable at the present time. Furthermore, the combination of established multicomponent reactions with post-reaction transformations opens the way towards a vast number of diverse and complex products. Some of these post-MCR transformations are: intramolecular cycloaddition reactions, Knoevenagel condensations, metathesis reactions, aza-Wittig reactions, Mitsunobu reactions, etc. [21].

Up to now, two review articles have been reported on azaheterocyclic phosphonates [22–23], but no overview article about the multicomponent synthesis of phosphono-substituted heterocycles has been reported so far. This review focuses on general multicomponent reactions as well as on modified MCR towards heterocyclic phosphonates. It is organized by the reaction types and covers literature published up to September 2015.

## Review

### 1 Biginelli condensation

**Scheme 1:**
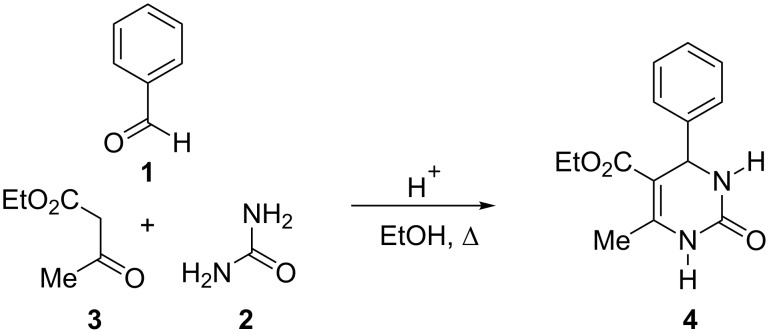
The Biginelli condensation.

The classical Biginelli condensation involves the reaction of an aldehyde **1** with urea (**2**) and a β-ketoester **3** under acidic conditions in refluxing ethanol to yield 3,4-dihydropyrimidin-2-one derivatives **4** (Scheme 1) [24].

**Scheme 2:**
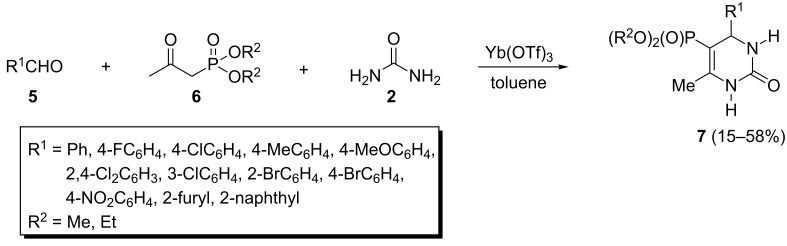
The Biginelli reaction of β-ketophosphonates catalyzed by ytterbium triflate.

Although, a large number of CH-acidic carbonyl compounds such as β-diketones, β-keto thioesters, acetoacetamides and nitroacetone have been shown to participate in the classical Biginelli reaction [25], β-ketophosphonates **6** were found to be unreactive in similar conditions [26]. However, Yuan et al. developed a modified Biginelli condensation by using ytterbium triflate as a catalyst (Scheme 2) [26] and the 3,4-dihydropyrimidin-2-one derivatives **4** were formed in 15–58% yields depending on the structure of the β-ketophosphonate **6** and aldehyde **5**. Based on their investigations, aliphatic aldehydes including propionaldehyde and butyraldehyde were resistant to this reaction.

**Scheme 3:**
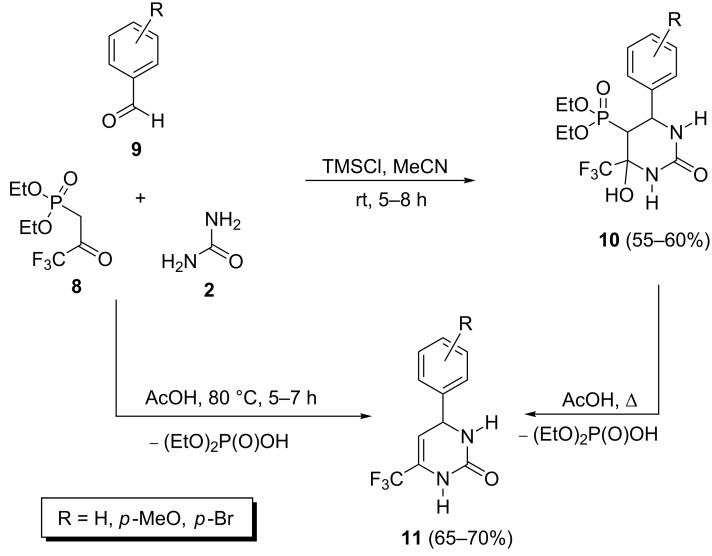
Trimethylchlorosilane-mediated Biginelli reaction of diethyl (3,3,3-trifluoropropyl-2-oxo)phosphonate.

The trimethylchlorosilane-mediated one-pot reaction of diethyl (3,3,3-trifluoropropyl-2-oxo)phosphonate (**8**) with aryl aldehydes **9** and urea under Biginelli conditions has been presented by Timoshenko et al. (Scheme 3) [27]. The resulting 4-hydroxytetrahydropyrimidin-2-ones **10** were unstable and underwent dephosphorylation to give dihydropyrimidin-2-ones **11** after one week at room temperature. Also, heating of either the reactants or product **10** in the presence of acetic acid led to the formation of dihydropyrimidin-2-ones **11** (Scheme 3).

**Scheme 4:**
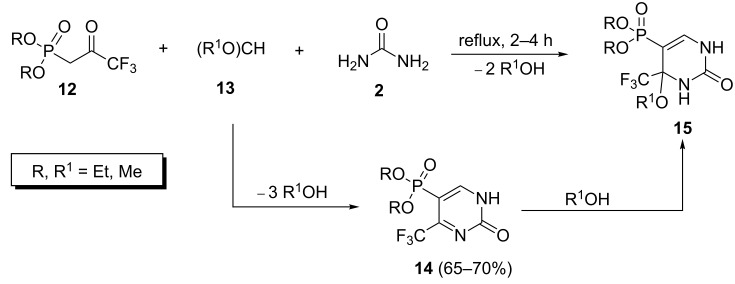
Biginelli reaction of dialkyl (3,3,3-trifluoropropyl-2-oxo)phosphonate with trialkyl orthoformates and urea.

However, the authors successfully used trialkyl orthoformates **13** to produce dialkyl (2-oxo-4-(trifluoromethyl)-1,2-dihydropyrimidin-5-yl)phosphonates **14** which were converted to dialkyl (4-alkoxy-2-oxo-4-(trifluoromethyl)-1,2,3,4-tetrahydropyrimidin-5-yl)phosphonates **15** through the nucleophilic addition of the liberated alcohol to the electrophilic double bond of the CF_3_−C=N segment (Scheme 4).

**Scheme 5:**
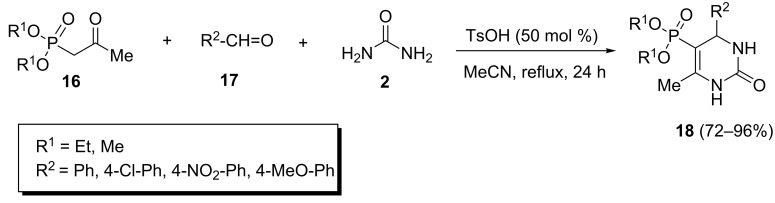
*p*-Toluenesulfonic acid-promoted Biginelli reaction of β-ketophosphonates, aryl aldehydes and urea.

Idris Essid and Soufiane Touil showed that the Biginelli condensation of β-ketophosphonates was highly sensitive to the nature of solvents, acid catalysts and reactants [28]. They found that the use of inorganic acids including HCl and H_2_SO_4_ or Lewis acids such as SnCl_2_, FeCl_3_ and VCl_3_, as well as heterogeneous catalysts including silica gel supported sulfuric acid and sodium hydrogen sulfate did not affect this reaction. Also, the reaction in the presence of *p*-toluenesulfonic acid (TsOH), in aprotic solvents proceeded with much better yields than in protic solvents. When diethyl (2-oxopropyl)phosphonate and 4-nitrobenzaldehyde were treated in the presence of 50 mol % TsOH in acetonitrile, 5-phosphonato-3,4-dihydropyrimidin-2-one **18** was obtained in excellent yield (Scheme 5).

### 2 Kabachnik−Fields reaction and its post-condensation modifications

**Scheme 6:**
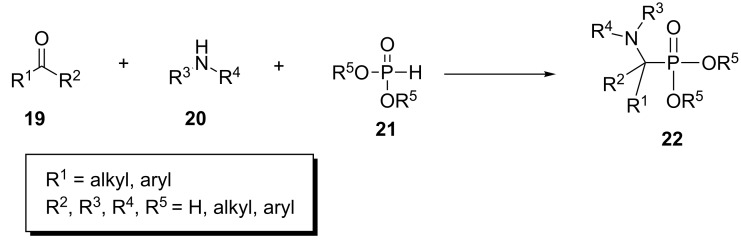
General Kabachnik–Fields reaction for the synthesis of α-aminophosphonates.

The one-pot three-component reaction between aldehydes **19** (or ketones), amines **20** and dialkyl phosphonates **21** to afford α-aminophosphonates **22** is traditionally known as the Kabachnik–Fields reaction. This reaction was first reported in 1952 by Kabachnik, Medved and Fields (Scheme 6) [29–30].

Due to their wide range of biological activities, α-aminophosphonates have been extensively investigated and several reviews about their syntheses through the Kabachnik–Fields reaction have been reported [31–33]. However, an important feature of this reaction is that it provides an efficient route to phosphonylated heterocycles. The different applications of the Kabachnik–Fields reaction in the preparation of phosphonylated heterocycles can be classified into two major categories: a) phosphonylation of heterocyclic ketones through a classic Kabachnik–Fields reaction and b) synthesis of heterocyclic phosphonates through modification of the products obtained by the Kabachnik–Fields reaction.

#### 2.1 Phosphorylation of the parent heterocycles through a traditional Kabachnik–Fields reaction

**Scheme 7:**
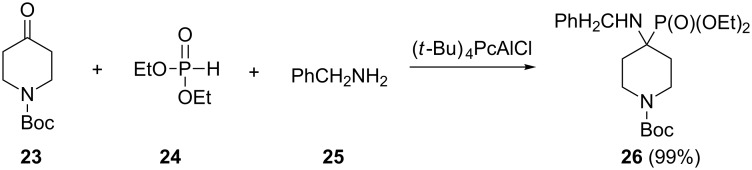
Phthalocyanine–AlCl catalyzed Kabachnik–Fields reaction of *N*-Boc-piperidin-4-one with diethyl phosphite and benzylamine.

Heterocycloalkanones may be used as carbonyl components in the Kabachnik–Fields reaction to give cyclic α-aminophosphonates. Unfortunately there are only a few examples of Kabachnik–Fields reactions of heterocycloalkanones in the literature. The tetra(*tert*-butyl)phthalocyanine–AlCl complex catalyzed three component reaction of *N*-Boc-piperidin-4-one (**23**) with (EtO)_2_P(O)H (**24**) and benzylamine (**25**) afforded the cyclic α-aminophosphonate **26** in 99% yield (Scheme 7) [34].

**Scheme 8:**
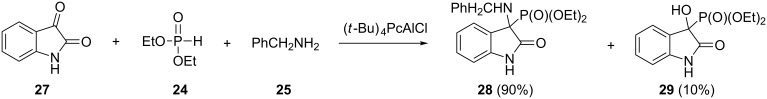
Kabachnik–Fields reaction of isatin with diethyl phosphite and benzylamine.

The reaction of isatin (**27**) with diethyl phosphite and benzylamine under similar conditions gave the corresponding α-aminophosphonate **28** in 90% yield together with small amounts of α-hydroxyphosphonate **29** as a side product (Scheme 8).

**Scheme 9:**
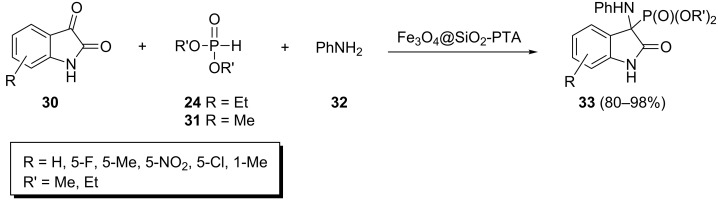
Magnetic Fe_3_O_4_ nanoparticle-supported phosphotungstic acid-catalyzed Kabachnik–Fields reaction of isatin with dialkyl phosphites and aniline.

The one-pot reaction of substituted isatins **30** with aniline (**32**) and dimethyl- or diethyl phosphite under solvent-free conditions in the presence of magnetic Fe_3_O_4_ nanoparticle-supported phosphotungstic acid as a recyclable catalyst at 80 °C furnished α-aminophosphonates **33** in yields from 80% to 98% depending on the reaction time and the structure of the dialkyl phosphite and isatin (Scheme 9) [35].

**Scheme 10:**
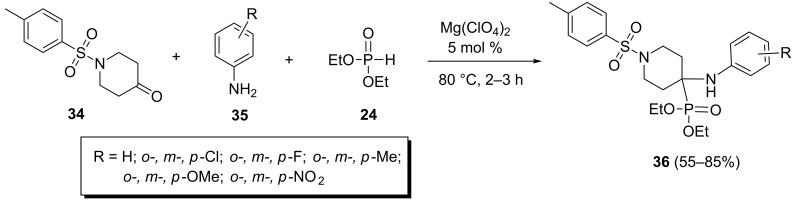
The Mg(ClO_4_)_2_-catalyzed Kabachnik–Fields reaction of 1-tosylpiperidine-4-one.

In this way a one-pot three-component reaction between 1-tosylpiperidine-4-one (**34**), aromatic amines **35** and diethyl phosphonate in the presence of magnesium perchlorate as a catalyst, under neat conditions at 80 °C afforded α-aminophosphonates **36** in good yields (Scheme 10). Some of the resulting α-aminophosphonates showed insecticidal activity against Plutella xylostella [36].

**Scheme 11:**
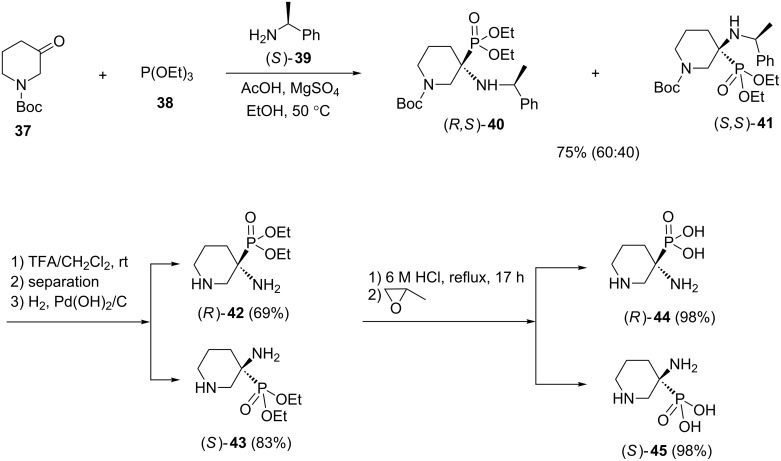
An asymmetric version of the Kabachnik–Fields reaction for the synthesis of α-amino-3-piperidinylphosphonic acids.

An asymmetric synthesis of heterocyclic α-aminophosphonates has been reported by Fadel et al. [37]. Their studies showed that the three-component reaction of *N*-Boc-3-piperidinone (**37**), (*S*)-configured amines **39** and triethyl phosphite (**38**), in the presence of 2 equiv of AcOH and 0.8 equiv of MgSO_4_ at 50 °C afforded a 60:40 diastereomeric mixture of α-aminophosphonates (*R*,*S*)-**40** and (*S*,*S*)-**41** in 75% combined yield. The cleavage of the *N*-Boc group followed by removal of the benzyl groups and acidic hydrolysis of the resulting (α-amino-3-piperidinyl)phosphonates (*R*)-**42** and (*S*)-**43** led to enantiopure α-amino-3-piperidinylphosphonic acids (*R*)-**44** and (*S*)-**45** in good yields (Scheme 11).

#### 2.2 Construction of heterocyclic phosphonates via post-condensation modification of Kabachnik–Fields reaction

**Scheme 12:**
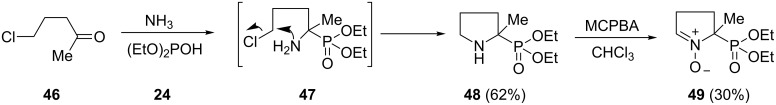
A classical Kabachnik–Fields reaction followed by an intramolecular ring-closing reaction for the synthesis of diethyl (2-methyl-2-pyrrolidinyl)phosphonate.

**2.2.1 2-Pyrrolidinylphosphonate:** Post-condensation modifications of Kabachnik–Fields reaction products usually rely on transformations of functional groups present in the formed α-aminophosphonates or by the use of additional reagents. One of the most important sequences for the rapid access of heterocyclic phosphonates is the combination of the Kabachnik–Fields reaction with a subsequent ring closure. Thus, the one-pot reaction of 5-chloro-2-pentanone (**46**) with ammonia and diethyl phosphonate in ethanol lead to the non-isolable intermediate **47** which was directly converted to diethyl (2-methyl-2-pyrrolidinyl)phosphonate **48** by ring closure through an intramolecular nucleophilic substitution (Scheme 12). Subsequent oxidation of **48** with *m*-chloroperbenzoic acid afforded the corresponding *N*-oxide **49** which was used for the in vitro and in vivo spin trapping of hydroxyl and superoxide radicals [38].

**Scheme 13:**
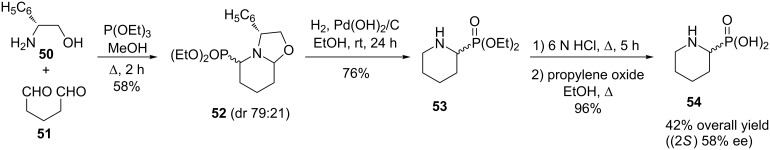
Synthesis of (*S*)-piperidin-2-phosphonic acid through an asymmetric Kabachnik–Fields reaction.

**2.2.2 2-Phosphono-6-oxazolopiperidines:** An asymmetric Kabachnik–Fields reaction between (*R*)-(−)-phenylglycinol (**50**), glutaraldehyde (**51**) and triethyl phosphite has been reported by Royer et al. The reaction furnished a diastereomeric mixture of 2-phosphono-6-oxazolopiperidines **52** with 58% yield and 79:21 dr. Further reduction and hydrogenolysis of **52** in the presence of Pd/C led to aminoester **53** which was converted to (*S*)-piperidin-2-phosphonic acid (**54**) through acidic hydrolysis and subsequent treatment with propylene oxide in 42% overall yield and 58% ee (Scheme 13) [39].

**Scheme 14:**
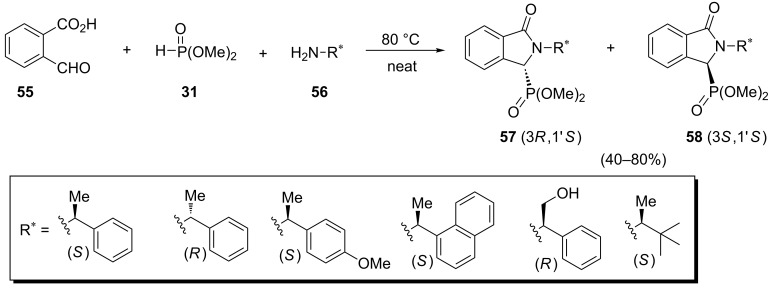
A modified diastereoselective Kabachnik–Fields reaction for the synthesis of isoindolin-1-one-3-phosphonates.

**2.2.3 Isoindolinylphosphonates:** A one-pot diastereoselective multicomponent reaction towards (isoindolin-1-one-3-yl)phosphonates has been developed by Ordóñez et al. (Scheme 14) [40]. The reaction of 2-formylbenzoic acid (**55**) with (*S*)- or (*R*)-amines **56** and dimethyl phosphonate under solvent and catalyst-free conditions at 80 °C afforded (3*R*,1'*S*)-isoindolin-1-one-3-phosphonates **57** and (3*S*,1'*S*)-isoindolin-1-one-3-phosphonates **58**. The best results concerning yield and selectivity were obtained with (*S*)-methylbenzylamine that furnished isoindolin-1-one-3-phosphonates **57** and **58** in 75% yields and a 95:05 diastereoisomeric ratio.

**Scheme 15:**
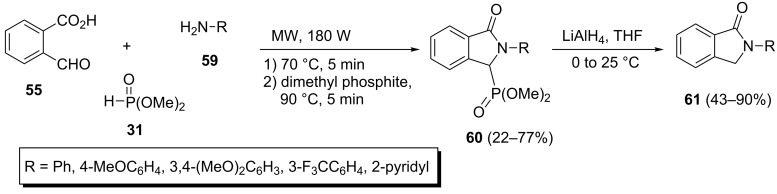
A microwave-assisted Kabachnik–Fields reaction toward isoindolin-1-ones.

The same research group found a useful method for the synthesis of isoindolin-1-one-3-phosphonates from aromatic amines [41]. The one-pot reaction of aniline **59** with formylbenzoic acid (**55**) and dimethyl phosphonate (**31**) under the above-mentioned conditions at 90 °C afforded the desired isoindolin-1-one-3-phosphonates **60** in only 14% yields after five days. Noteworthy, the treatment of the same reaction mixture under microwave irradiation at 90 °C gave the expected product **60** in 77% yields after several minutes. Subsequently, the isoindolin-1-one-3-phosphonates **60** were dephosphorylated by treatment with lithium aluminum hydride to give isoindolin-1-ones **61** (Scheme 15).

**Scheme 16:**
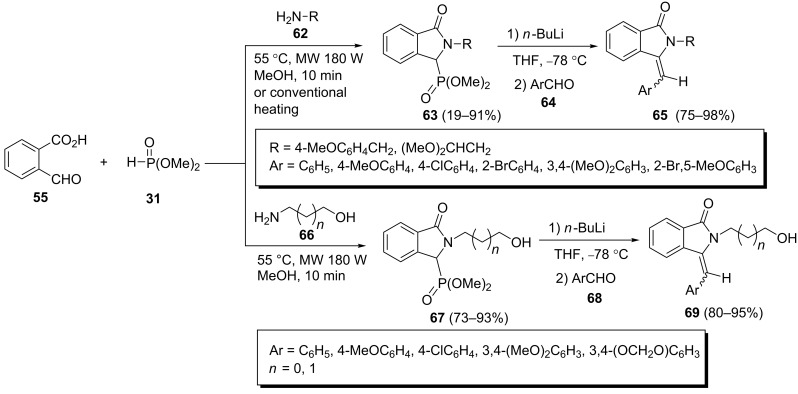
The synthesis of 3-arylmethyleneisoindolin-1-ones through a Horner–Wadsworth–Emmons reaction of Kabachnik–Fields reaction products.

Also the Kabachnik–Fields reaction of formylbenzoic acid (**55**), dimethyl phosphonate and amines **62** or **66** followed by subsequent Horner–Wadsworth–Emmons reaction of the resulting cycloadducts **63** and **67** with arylaldehydes **64** or **68** afforded the corresponding 3-arylmethyleneisoindolin-1-ones **65** and **69**, respectively (Scheme 16) [42–43].

**Scheme 17:**
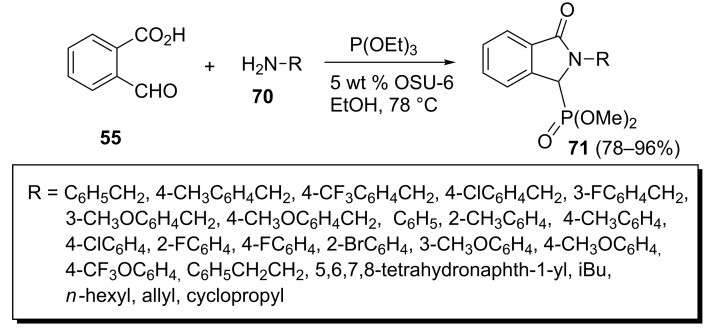
An efficient one-pot method for the synthesis of ethyl (2-alkyl- and 2-aryl-3-oxoisoindolin-1-yl)phosphonates.

Recently, an efficient method was developed for the synthesis of ethyl (2-alkyl- and 2-aryl-3-oxoisoindolin-1-yl)phosphonates **71** from 2-formylbenzoic acid (**55**), triethyl phosphite and amines **70** using OSU-6, a novel MCM-41-type hexagonal mesoporous silica, as a catalyst (Scheme 17) [44]. The important advantages of this methodology is that the (3-oxoisoindolin-1-yl)phosphonates **71** are obtained in high yields from benzylic, aliphatic and aromatic amines possessing both, electron-donating and electron-withdrawing groups, in shorter reaction times with minimum purification requirements. Also, the catalyst can be used for up to four reaction cycles without significant loss of activity.

**Scheme 18:**
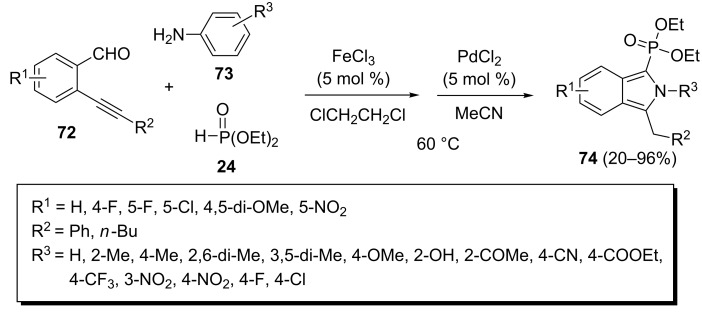
FeCl_3_ and PdCl_2_ co-catalyzed three-component reaction of 2-alkynylbenzaldehydes, anilines, and diethyl phosphonate.

An efficient method for the synthetic preparation of diverse (2*H*-isoindol-1-yl)phosphonates **74** is an FeCl_3_-catalyzed Kabachnik–Fields reaction of 2-alkynylbenzaldehydes **72**, anilines **73**, and phosphonates followed by a PdCl_2_-catalyzed 5-*exo*-*dig* cyclization (Scheme 18) [45]. The desired (2*H*-isoindol-1-yl)phosphonates **74** were obtained under optimized conditions (5 mol % of FeCl_3_, 5 mol % of PdCl_2_, DCE/CH_3_CN, 60 °C) in good to excellent yields. One limitation is the use of aromatic aldehydes bearing electron-donating substituents which afforded the desired products in only low yields, because of their reduced electrophilicity. Aliphatic amines were unreactive in this transformation and only arylamines were found to be effective in this reaction. Eventually, the biological evaluation of the (2*H*-isoindol-1-yl)phosphonates **74** revealed their potential as HCT-116 inhibitors.

**Scheme 19:**
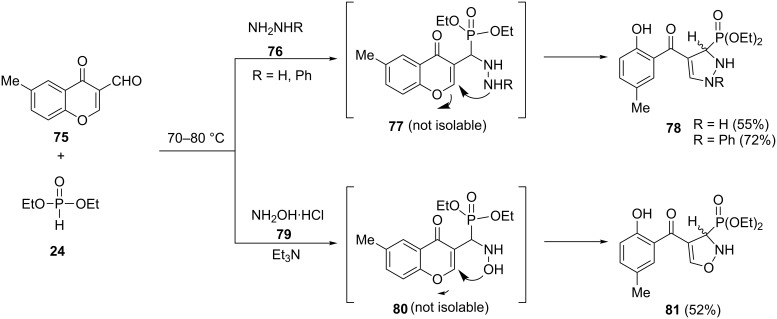
Three-component reaction of 6-methyl-3-formylchromone (**75**) with hydrazine derivatives or hydroxylamine in the presence of diethyl phosphonate.

**2.2.4 Pyrazolyl- and oxazolylphosphonates:** A series of modified Kabachnik–Fields condensations based on the reaction of 6-methyl-3-formylchromone (**75**) with some 1,2-, 1,3- and 1,4-bi-nucleophiles and diethyl phosphonate under solvent-free conditions have been developed by E. Ali et al. [46]. The resulting α-aminophosphonate intermediates **77** and **80** were non-isolable and interconverted to the corresponding heterocyclic phosphonates via ring opening through an intramolecular nucleophilic attack at the 2-position of the pyrone. Thus, the three-component reaction of **75** with hydrazine derivatives **76** or hydroxylamine **79** in the presence of diethyl phosphonate led to pyrazolylphosphonate **78** and oxazolylphosphonate **81**, respectively (Scheme 19).

**Scheme 20:**
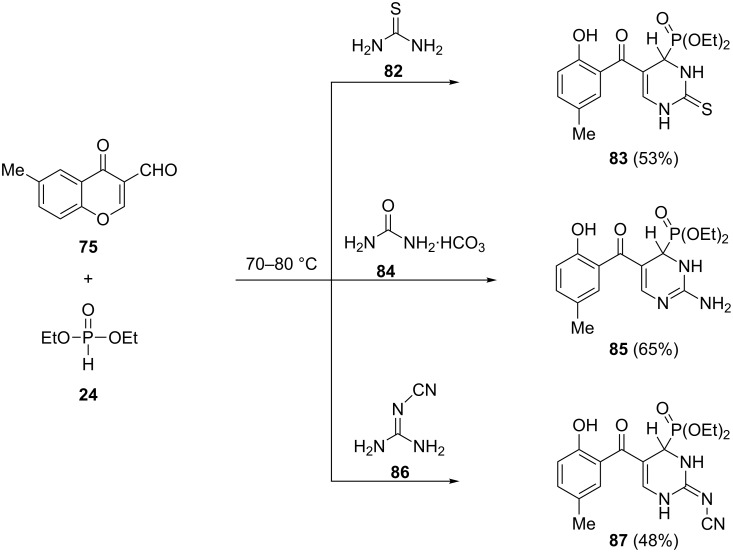
Three-component reaction of 6-methyl-3-formylchromone (**75**) with thiourea, guanidinium carbonate or cyanoguanidine in the presence of diethyl phosphonate.

**2.2.5 Pyrimidinylphosphonates:** The 1,3-bi-nucleuphiles such as thiourea (**82**), guanidinium carbonate **84** and cyanoguanidine **86**, under the above mentioned conditions afforded the pyrimidinylphosphonates **83**, **85** and **87**, respectively (Scheme 20).

**Scheme 21:**
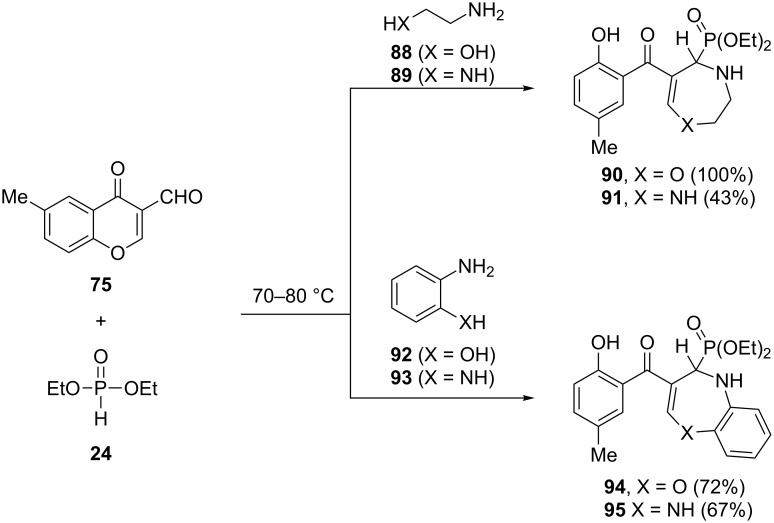
Three-component reaction of 6-methyl-3-formylchromone (**75**) with 1,4-bi-nucleophiles in the presence of diethyl phosphonate.

**2.2.6 Diazepinyl- and oxazepinylphosphonates:** The three-component reaction of 1,4-bi-nucleophiles such as ethanolamine (**88**), ethylenediamine (**89**), 2-aminophenol (**92**) and 1,2-phenylenediamine (**93**), with 6-methyl-3-formylchromone (**75**) and diethyl phosphonate afforded the phosphonate derivatives of 1,4-oxazepine **90**, 1,4-diazepine **91**, 1,5-benzoxazepine **94** and 1,5-benzodiazepine **95**, respectively (Scheme 21).

**Scheme 22:**
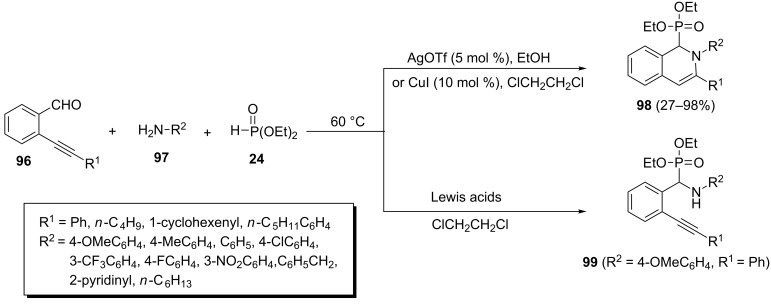
One-pot three-component reaction of 2-alkynylbenzaldehydes, amines, and diethyl phosphonate.

**2.2.7 Isoquinolone-1-phosphonates:** From Lewis acid catalyzed 6-*endo-dig* cyclizations of acetylenic Kabachnik–Fields adducts: A modified Kabachnik–Fields reaction for the synthesis of isoquinoline-1-phosphonate derivatives is the three-component reaction of acetylenic aldehydes with various amines and dialkyl phosphonates followed by Lewis acid catalyzed 6*-endo-dig* cyclizations. Wu et al. reported the one-pot reaction of 2-alkynylbenzaldehydes **96**, amines **97**, and diethyl phosphonate to afford (2,3-disubstituted-1,2-dihydroisoquinolin-1-yl)phosphonates **98** in the presence of various Lewis acids (Scheme 22) [47–48]. This reaction, under catalyst-free conditions or in the presence of Lewis acids such as FeCl_3_, CBr_4_, In(OTf)_3_, Bi(OTf)_3_, and Yb(OTf)_3,_ exclusively yielded the acyclic α-aminophosphonates **99**. However, the reaction in the presence of AgOTf (5 mol %) or CuI (10 mol %) at 60 °C led to isoquinolin-1-ylphosphonates **98** in moderate to high yields.

In addition, this reaction in the presence of palladium catalysts such as Pd(PPh_3_)_2_Cl_2_ and PdCl_2_ gave 1,2-dihydroisoquinolin-1-ylphosphonates **98** in 79% and 81% yields, respectively.

**Scheme 23:**
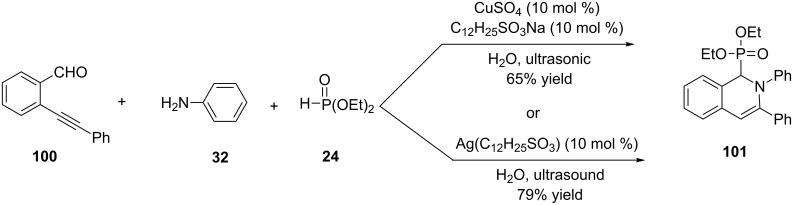
Lewis acid–surfactant combined catalysts for the one-pot three-component reaction of 2-alkynylbenzaldehydes, amines, and diethyl phosphonate.

Lewis acid–surfactant combined catalysts (LASC) are another catalytic system which has been used for the three-component reaction of 2-alkynylbenzaldehydes, amines, and nucleophiles such as alkynes, nitromethane, or diethyl phosphonate in water under ultrasonic conditions [49]. As depicted in Scheme 23, the reaction of 2-alkynylbenzaldehyde **100**, aniline (**32**) and diethyl phosphonate catalyzed by C_12_H_25_SO_3_Na–CuSO_4_ (10 mol %) or Ag(C_12_H_25_SO_3_) (10 mol %) under ultrasonic conditions in an aqueous medium afforded the desired 1,2-dihydroisoquinolin-1-ylphosphonate **101** in 65% and 79% yields, respectively.

**Scheme 24:**
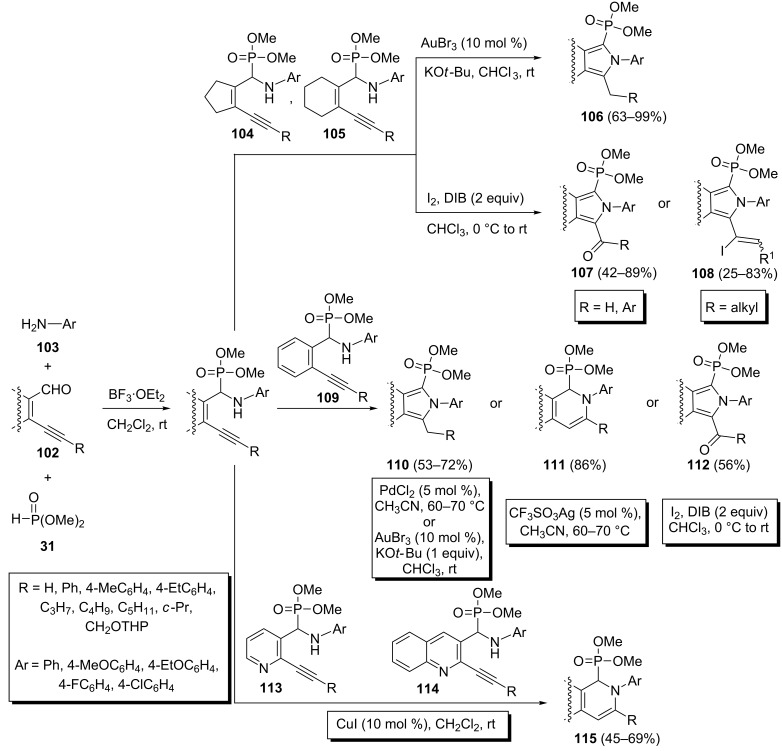
Lewis acid catalyzed cyclization of different Kabachnik–Fields adducts.

A more detailed investigation on the catalytic cyclization during Kabachnik–Fields reactions of acetylenic aldehydes with aromatic amines and dialkyl phosphonates has been reported by Čikotienė et al. [50]. They found that the cyclization type during these three-component reactions strongly depends on the nature of the acetylenic aldehydes **102**. The Kabachnik–Fields adducts of various carbocyclic acetylenic aldehydes **104** and **105** in the presence of AuBr_3_, PdCl_2_, AgOTf, AgNO_3_ or I^+^ underwent a 5*-exo-dig* cyclization to give dialkyl 1*H*-pyrrol-2-ylphosphonates **106**. However, iodine-mediated cyclizations lead to pyrrol-1-ylphosphonates bearing a carbonyl (**107**) or 1-iodoalkenyl substituent (**108**) depending on the substituent R. In contrast, electron-deficient heterocycles **113** and **114** in the presence of CuI reacted through a tandem imine formation–6*-endo-dig* cyclization to give the corresponding 1,2-dihydropyridin-2-ylphosphonates **115**. However, electron-rich heterocyclic Kabachnik–Fields adducts were found to be unreactive towards Lewis acid catalyzed cyclization processes. On the other hand, benzene derivatives **109** can participate in both cyclization modes depending on the catalyst’s nature. They either can cyclize to give the corresponding 1,2-dihydropyridin-2-ylphosphonates **111** in the presence of CF_3_SO_3_Ag, while in the presence of AuBr_3_, PdCl_2_ or I^+^, they undergo a 5*-exo-dig* cyclization to give dialkyl 1*H*-pyrrol-2-ylphosphonates **110** or **112** (Scheme 24).

**Scheme 25:**
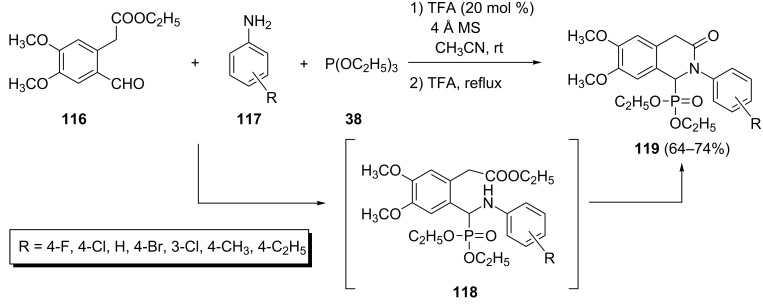
Three-component synthesis of *N*-arylisoquinolone-1-phosphonates **119**.

Other cyclization reactions of Kabachnik–Fields adducts into isoquinolone-1-phosphonates: A one-pot three-component synthesis of *N*-arylisoquinolone-1-phosphonates **119** through the Kabachnik–Fields reaction of ethyl 2-(2-formyl-4,5-dimethoxyphenyl)acetate (**116**) with anilines **117** and triethyl phosphite in the presence of trifluoroacetic acid as catalyst has been reported by Borse et al. (Scheme 25) [51]. The desired *N*-arylisoquinolone-1-phosphonates **119** were formed through the intramolecular addition of the amino group to the ester functionality in the Kabachnik–Fields adducts **118**. The yields ranged between 64% and 74% depending on the nature of the substituents present in the aromatic amines **117**.

**Scheme 26:**
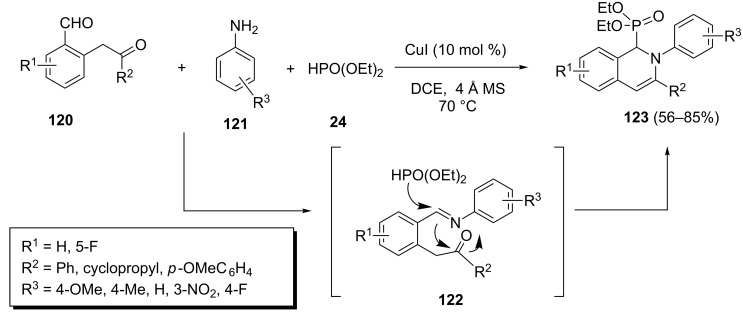
CuI-catalyzed three-component tandem reaction of 2-(2-formylphenyl)ethanones with aromatic amines and diethyl phosphonate.

A CuI-catalyzed three-component tandem reaction of 2-(2-formylphenyl)ethanones **120**, aromatic amines **121**, and diethyl phosphonate leading to 1,2-dihydroisoquinolin-1-ylphosphonates **123** has been reported by Wu et al. (Scheme 26) [52]. This reaction proceeds via the imine intermediate **122** resulting from the reaction of 2-(2-formylphenyl)ethanones **120** with amines **121**. The tandem nucleophilic addition of phosphite to the imine and subsequent condensation of the formed amine with the ketone group leads to 1,2-dihydroisoquinolin-1-ylphosphonates **123**. A wide range of substituted aromatic amines and several 2-(2-formylphenyl)ethanones under optimized conditions [CuI (10 mol %), 1,2-dichloroethane, 70 °C] afforded the corresponding 1,2-dihydroisoquinolin-1-ylphosphonates in good to excellent yields.

**Scheme 27:**
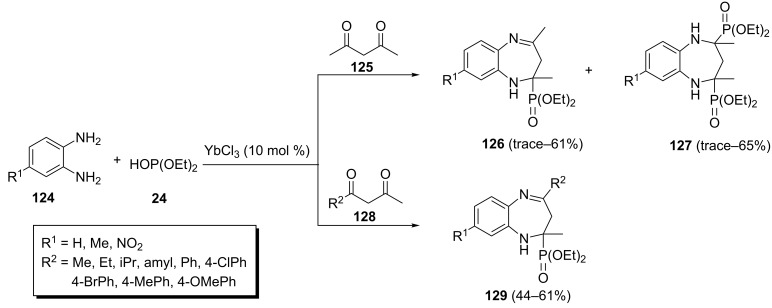
Synthesis of 1,5-benzodiazepin-2-ylphosphonates via ytterbium chloride-catalyzed three-component reaction.

**2.2.8 Benzodiazepinylphosphonates:** There are only two publications related to the multicomponent synthesis of benzodiazepinylphosphonates in the literature. Both syntheses are based on *o*-diaminobenzene as the starting material. In the first method, an YbCl_3_-catalyzed three-component reaction between an *o*-diaminobenzene **124**, 2,4-pentanedione (**125**) and diethyl phosphite under optimized solvent-free conditions (10 mol % of YbCl_3_, 22 °C, 1:1:1 molar ratio of starting materials) afforded 1,5-benzodiazepin-2-ylphosphonates **126** in moderate yields (Scheme 27) [53]. The undesired diphosphonate **127** was formed in 1:1:2 or 1:1:4 molar ratio of *o*-diaminobenzene **124**, 1,3-butanediones **125** and diethyl phosphite. When butanediones **128** with a larger substituent than Me were used, only monophosphonates **129** were obtained.

**Scheme 28:**
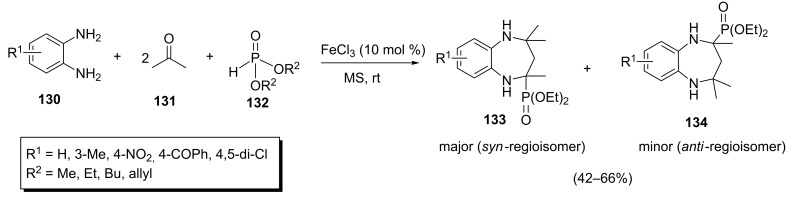
FeCl_3_-catalyzed four-component reaction for the synthesis of 1,5-benzodiazepin-2-ylphosphonates.

The second method comprises a one-pot four-component reaction of diamines **130**, ketones **131** and phosphites **132** in the presence of FeCl_3_ as a catalyst to give benzodiazepinylphosphonates **133** and **134** and has been reported by Bhattacharya et al. (Scheme 28) [54]. The authors observed that the presence of molecular sieves (4 Å) had a beneficial effect on the yield of the reaction due to trapping of water resulting from the imine formation reaction. The generality of the reaction has been investigated by the use of structurally diverse diamines, ketones and phosphonates. While the reaction proceeded well with different amines and phosphonates, only the use of acetone as the ketone component afforded the corresponding benzodiazepinylphosphonates. With other ketones only ketimine intermediates were obtained which were sterically too crowded for attacking the phosphorus atom of the phosphonates. The use of unsymmetrically substituted diamines led to the corresponding syn-regioisomers as the major product and the anti-regioisomer as the minor product. Some of the synthesized 1,5-benzodiazepin-2-ylphosphonates showed cysteine protease inhibition activities.

**Scheme 29:**
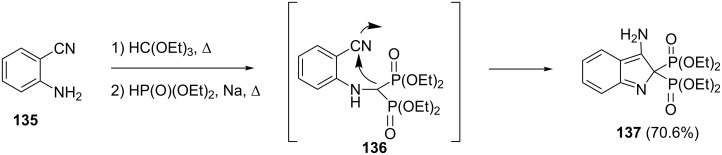
Synthesis of indole bisphosphonates through a modified Kabachnik–Fields reaction.

**2.2.9 Heterocyclic bisphosphonates:** A modified Kabachnik–Fields reaction of the substituted amine **135** with triethyl orthoformate followed by reaction with sodium diethylphosphite afforded bisphosphonate intermediate **136** that was converted to the heterocyclic bisphosphonate **137** through an intramolecular cyclization (Scheme 29) [55]. The sequenced reaction of the amine with triethyl orthoformate followed by the addition of sodium diethylphosphite dissolved in toluene considerably increased the yields of bisphosphonates.

**Scheme 30:**
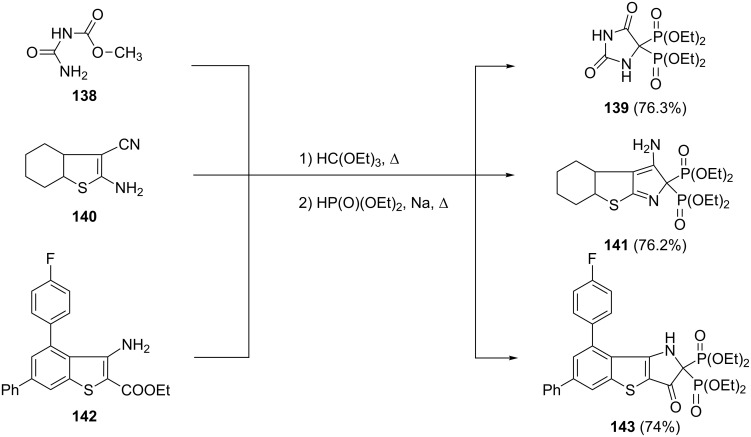
Synthesis of heterocyclic bisphosphonates via Kabachnik–Fields reaction of triethyl orthoformate.

In this way, carbamate **138**, hexahydrobenzothiophene **140** and benzothiophene **142** were converted to the corresponding bisphosphonates **139**, **141** and **143**, respectively (Scheme 30). The synthesized heterocyclic bisphosphonates showed anti-inflammatory properties.

### 3 Knoevenagel-induced domino reactions

An efficient method into phosphorylated heterocycles is the condensation of an activated methylene component with a carbonyl compound followed by subsequent transformations such as intramolecular cyclization, Michael-type addition and hetero-Diels–Alder cycloaddition.

#### 3.1 Domino Knoevenagel/phospha-Michael process

**Scheme 31:**
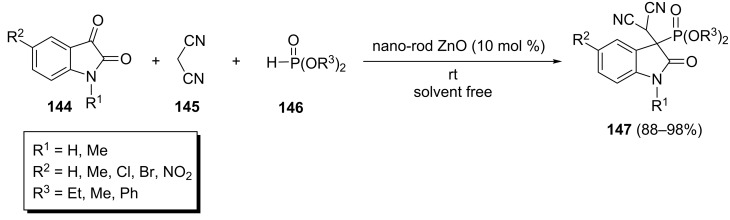
A domino Knoevenagel/phospha-Michael process for the synthesis of 2-oxoindolin-3-ylphosphonates.

A convenient one-pot ZnO nanorods-catalyzed reaction of isatin derivatives **144** with malononitrile (**145**) and dialkyl or diphenyl phosphonates **146** has been performed to give 2-oxindolin-3-ylphosphonates **147** (Scheme 31) [56]. The products were obtained in good to excellent yields using 10 mol % of the catalyst under solvent-free conditions at room temperature. However, when using ethyl cyanomalonate instead of malononitrile, the reaction in water led to the corresponding 2-oxoindolin-3-ylphosphonate in good yield. Further, the investigations showed that the recovered ZnO nanorods could be reused up to five times.

**Scheme 32:**
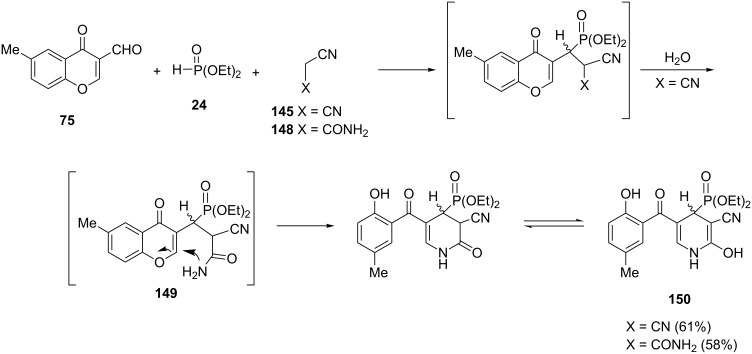
Intramolecular cyclization of phospha-Michael adducts to give dihydropyridinylphosphonates.

Some of the phospha-Michael adducts were converted to new phosphorylated heterocycles through intramolecular cyclization reactions. The phospha-Michael adduct **149** resulting from the three-component reaction of 6-methyl-3-formylchromone with malononitrile **145** or 2-cyanoacetamide **148** and diethyl phosphite is not isolable and spontaneously recyclized to dihydropyridinylphosphonate **150** (Scheme 32) [46].

**Scheme 33:**
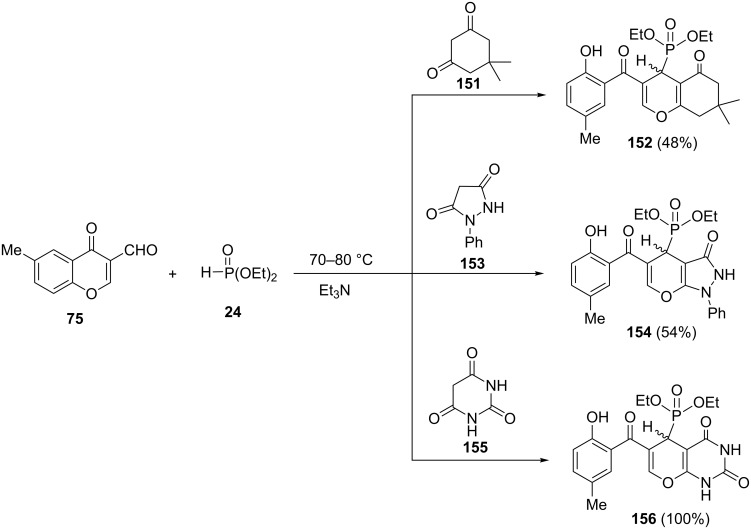
Synthesis of fused phosphonylpyrans via intramolecular cyclization of phospha-Michael adducts.

In this way, the reaction of 6-methyl-3-formylchromone (**75**) with cyclic 1,3-dicarbonyl compounds such as dimedone (**151**), 1-phenylpyrazolidine-3,5-dione (**153**) or barbituric acid (**155**) afforded the fused phosphonylpyrans **152**, **154** and **156**, respectively (Scheme 33).

#### 3.2 Three-component synthesis of (2-amino-3-cyano-4*H*-chromen-4-yl)phosphonates

**Scheme 34:**
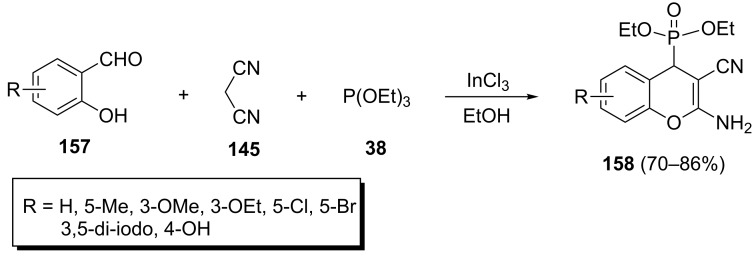
InCl_3_-catalyzed three-component synthesis of (2-amino-3-cyano-4*H*-chromen-4-yl)phosphonates.

Because of the widespread biological activities related to 2-amino-4*H*-chromene derivatives, the synthesis of (2-amino-3-cyano-4*H*-chromen-4-yl)phosphonates has attracted much attention from organic chemists. The best procedure for the preparation of these compounds involves a one-pot three-component reaction between salicylaldehydes **157**, malononitrile (**145**) and trialkyl phosphite that was first reported by Perumal et al. (Scheme 34) [57]. The desired (2-amino-3-cyano-4*H*-chromen-4-yl)phosphonates **158** were obtained in good yields in the presence of InCl_3_ (20 mol %) at room temperature in ethanol.

In recent years, several methods using different catalysts have been developed to prepare 2-amino-3-cyano-4*H*-chromen-4-ylphosphonates. These methods and other aspects of reaction conditions are summarized in Table 1.

**Table 1:** Different catalytical methods for the synthesis of 2-amino-3-cyano-4*H*-chromen-4-ylphosphonates.

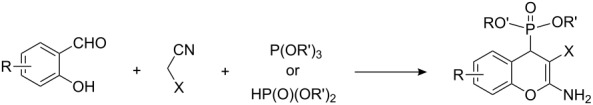							

Entry	R	R′	X	Catalyst	*T* (°C)	Yields (%)	Ref.

1	H, 3,5-di-Cl, 5-Br, 3,5-di-Br, 3,5-di-I, 3-OMe	Et	CN, COOEt	β-CD	60–70	76–88	[58]
2	H, 5-Cl, 3,5-di-Cl, 5-Br, 3,5-di-Br, 5-Me, 3-OMe, 5-OMe, 5-NO_2_, 5,6-(CH=CH)_2_	Et	CN	K_3_PO_4_	rt	74–95	[59]
3	H, 5-Cl, 3,5-di-Cl, 3,5-di-Br, 5-Me, 3-OMe, 4-Et_2_N, 4,5-(CH=CH)_2_	Et, Me	CN, COOEt	PEG-400	80	81–92	[60]
4	H, 5-Cl	Et	CN, COOEt	I_2_	rt	79–91	[61]
5	H, 5-Br, 3,5-di-Br, 3-Me, 5-Me, 4-OMe, 5-OMe, 5-NO_2_, 5,6-(CH=CH)_2_	Et	CN, COOEt	EDDA	rt	40–90	[62]
6	H, 5-Cl, 3,5-di-Cl, 5-Br, 3,5-di-Br, 3-Me, 5-OMe, 5-NO_2_	Et	CN	Et_2_NH	rt	90–95	[63]
7	H, 5-Cl, 5-Br, 5-Me, 3-OMe, 3-OEt	Et	CN	electrocatalysis	20–78	88–93	[64]
8	H, 5-Cl, 5-Br, 3-Me, 5-NO_2_, 3-*t*-Bu	Et, Me, Bu, Ph	CN, COOEt	TMG	rt	65–96	[65]
9	H, 5-Cl, 5-Br, 3,5-di-Cl, 3,5-di-Br, 5-Me, 5-OMe, 5-NO_2_	Et	CN, COOEt	nano-MgO	rt	68–92	[66]
10	H, 5-Br, 3,5-di-Cl, 3,5-di-Br, 3-Me, 5-OMe, 5-*t*-Bu, 3,5-di-*t*-Bu, 5,6-(CH=CH)_2_	Et, Me, iPr	CN	catalyst-free	rt	68–90	[67]
11	H, 5-Br, 5-NO_2,_ 3-*t*-Bu	Et, Me, Bu, iPr	CN	dibutylamine	rt	85–96	[68]
12	H, 5-Cl, 5-Br, 5-Me, 5-NO_2,_ 3,5-di-Br, 3-OMe, 3-OEt	Et, Ph	CN	LiOH	rt	85–97	[69]
13	H, 5-Cl, 5-Br, 5-Me, 3-Br, 3-OMe, 3-OEt	Et, Me, iPr	CN, COOEt	silica-bonded 2-HEAA	rt	71–87	[70]
14	H, 5-Br, 5-Cl, 3,5-di-Br, 3,5-di-Cl, 5-Me, 4-OMe, 5-OMe, 5-NO_2_	Et	CN	Fe_3_O_4_@CS-SO_3_H NPs	rt	88–97	[71]

#### 3.2 Domino Knoevenagel/hetero-Diels–Alder process

**Scheme 35:**
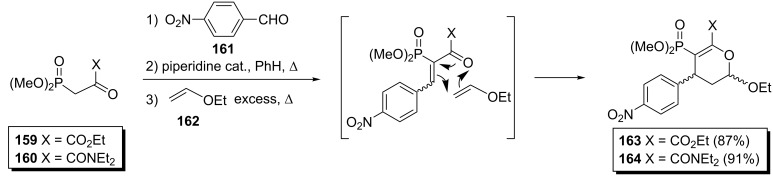
Synthesis of phosphonodihydropyrans via a domino Knoevenagel/hetero-Diels–Alder process.

The one-pot synthesis of dihydropyrans via a three-component reaction between an activated methylene compound, an aldehyde and an electron-rich alkene was firstly reported by Tietze et al. [72]. Collignon et al. applied this protocol for the preparation of phosphonodihydropyrans **163** or **164** starting from phosphonopyruvate **159** or phosphonopyruvamide **160**, *p*-nitrobenzaldehyde (**161**) and ethyl vinyl ether (**162**) in a reactor equipped with a Dean–Stark separator (Scheme 35) [73]. The yields of the resulting cycloadducts **163** and **164** were 87% and 91%, respectively and were much higher than the overall yields of the corresponding multi-step reactions. Also, the trans/cis selectivity of phosphonodihydropyrans **163** and **164** was 24:76 and 22:78, respectively.

**Scheme 36:**
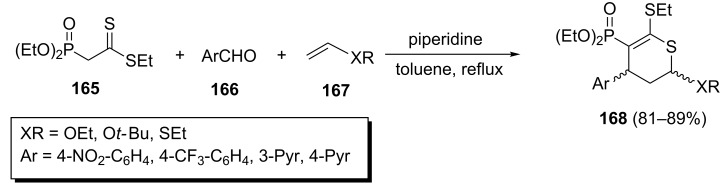
Multicomponent synthesis of phosphonodihydrothiopyrans via a domino Knoevenagel/hetero-Diels–Alder process.

In this way, Gulea et al. synthesized phosphonodihydrothiopyrans **168** through the one-pot reaction of phosphonodithioacetate **165**, aromatic aldehydes **166** and dienophiles **167** in the presence of piperidine in refluxing toluene (Scheme 36) [74]. The new phosphorylated cycloadducts were isolated in excellent yields and with a *trans*- or *cis*-diastereoselectivity.

### 4 Metal-catalyzed multicomponent reactions

Transition metals, especially Pd and Cu, are well-known catalysts for multicomponent reactions. Carbopalladation reactions of allenes, alkynes and carbon monoxide are very important processes in multicomponent syntheses. Additionally, copper-catalyzed multicomponent reactions such as azide–alkyne cycloadditions and various A^3^-coupling reactions are useful procedures in heterocyclic chemistry. However, several methods based on these protocols have also been developed for the synthesis of heterocyclic phosphonates.

**Scheme 37:**
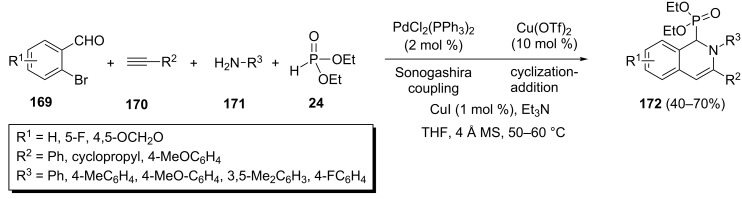
One-pot four-component synthesis of 1,2-dihydroisoquinolin-1-ylphosphonates under multicatalytic conditions.

The 1,2-dihydroisoquinolin-1-ylphosphonates **172** were formed through a one-pot reaction of 2-bromobenzaldehydes **169**, alkynes **170**, amines **171**, and diethyl phosphonate under multicatalytic conditions including palladium and copper salts (Scheme 37) [75]. This process presumably involves a sequential Sonogashira coupling/cyclization-nucleophilic addition reaction, which is catalyzed by PdCl_2_(PPh_3_)_2_ and CuI whereas Cu(OTf)_2_ acts as a Lewis acid. The desired 1,2-dihydroisoquinolin-1-ylphosphonates **169** were isolated under optimized conditions [PdCl_2_(PPh_3_)_2_ (2 mol %), CuI (1 mol %), Cu(OTf)_2_ (10 mol %), Et_3_N, 4 Å molecular sieves, THF, 50–60 °C] in 40–70% yields.

**Scheme 38:**
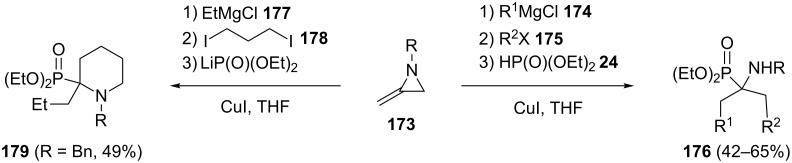
CuI-catalyzed four-component reactions of methyleneaziridines towards alkylphosphonates.

A CuI-catalyzed four-component reaction through a methyleneaziridine ring-opening process has been developed for the synthesis of α-aminophosphonates [76]. Thus, the one-pot reaction between methyleneaziridines **173**, Grignard reagents **174**, alkyl halides **175** and dialkyl phosphonates in the presence of CuI afforded acyclic α-aminophosphonates **176**. However, using difunctionalized electrophiles such as 1,3-diiodopropane **178** resulted in piperidinylphosphonates **179** with moderate yields (Scheme 38). This one-pot transformation involves an aziridine ring opening, C-alkylation, and hydrophosphorylation of the formed imine to create three intermolecular bonds.

**Scheme 39:**
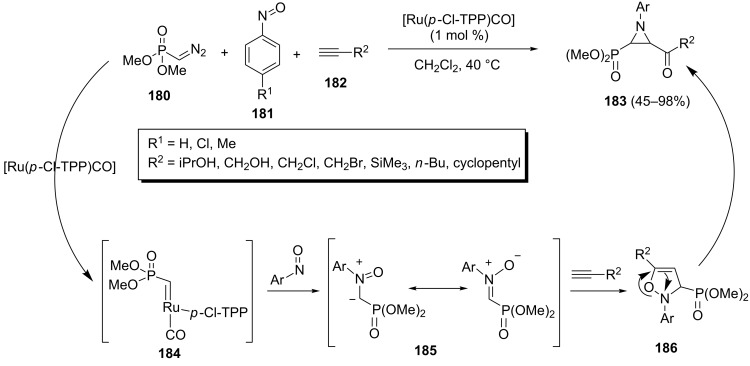
Ruthenium–porphyrin complex-catalyzed three-component synthesis of aziridinylphosphonates and its proposed mechanism.

A ruthenium–porphyrin complex-catalyzed three-component reaction of α-diazophosphonates **180**, nitrosoarenes **181**, and alkynes **182** to give multifunctionalized aziridinylphosphonates **183** has been reported by Reddy et al. (Scheme 39) [77]. The desired aziridinylphosphonates **183** were isolated in 45–98% yields and 90:10 to >99:1 diastereoisomeric ratio depending on the structure of substituents present on nitrosoarenes **181** and alkynes **182**. The use of internal alkynes gave only poor yields of the corresponding aziridinylphosphonates due to their low reactivity. This process involves the 1,3-dipolar cycloaddition of alkynes **182** with in situ generated nitrone **185** to afford isoxazolines **186** which rapidly rearrange to aziridinylphosphonates **183**.

**Scheme 40:**
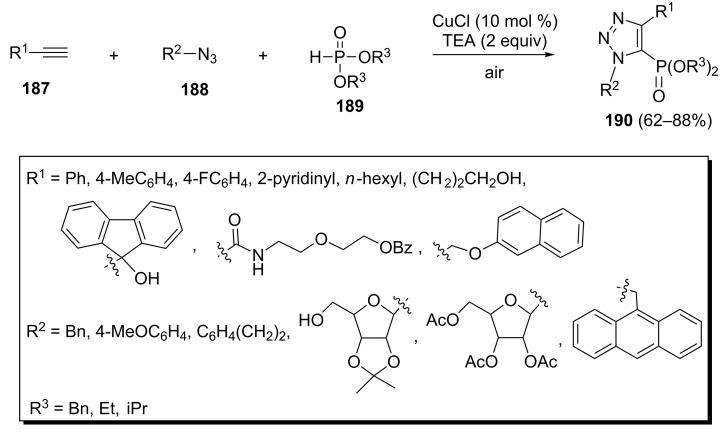
Copper(I)-catalyzed three-component reaction towards 1,2,3-triazolyl-5-phosphonates.

An efficient method for the synthesis of 1,2,3-triazoles is the copper(I)-catalyzed Husigen cycloaddition of azides with alkynes. Based on this method, Li et al. have developed a copper(I)-catalyzed three-component reaction between alkynes **187**, azides **188** and dialkyl phosphonates **189** to give 1,2,3-triazolyl-5-phosphonates **190** (Scheme 40) [78]. The desired products were obtained in 62–88% yield under optimized conditions [CuCl (0.015 mmol), TEA (0.3 mmol), MeCN, rt, 20 h].

### 5 Isocyanide-based multicomponent reactions

Although isocyanide-based multicomponent reactions (IMCRs) are one of the most important routes into heterocyclic compounds, there are only a few publications related to the isocyanide-based multicomponent synthesis of heterocyclic phosphonates in the literature. However, three different isocyanide-based multicomponent reactions for the synthesis of heterocyclic phosphonates are described here.

**Scheme 41:**
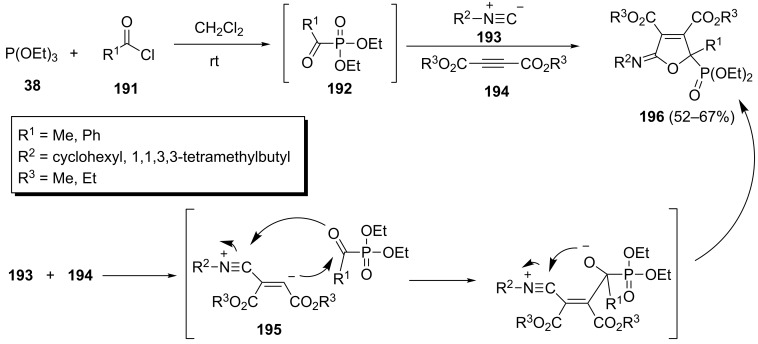
Three-component reaction of acylphosphonates, isocyanides and dialkyl acetylenedicarboxylate to afford 2-phosphonofuran derivatives.

A one-pot three-component reaction between the acylphosphonates **192** formed by treatment of triethyl phosphite and acyl chlorides **191**, isocyanides **193** and dialkyl acetylenedicarboxylates **194** to afford 2-phosphonofuran derivatives **196** has been reported by our group (Scheme 41) [79]. The desired furanylphosphonates were isolated in 52–67% yield at rt in CH_2_Cl_2_. In this transformation the zwitterionic intermediate **195**, resulting from reaction of isocyanide with dialkyl acetylenedicarboxylate, added to the carbonyl group of the acylphosphonate followed by an intramolecular cyclization.

**Scheme 42:**
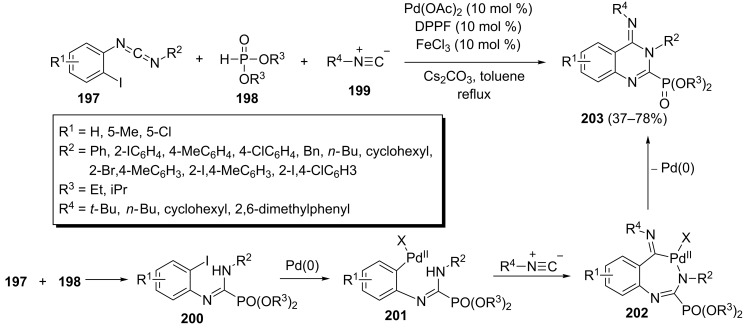
Synthesis of (4-imino-3,4-dihydroquinazolin-2-yl)phosphonates via an isocyanide-based three-component reaction.

A palladium-catalyzed isocyanide-based three-component pathway into phosphorylated quinazolines has been described by Wu et al. [80]. The one-pot reaction of carbodiimide **197**, isocyanide **199** and dialkyl phosphonates **198** under optimized conditions (10 mol % of Pd(OAc)_2_, 10 mol % of FeCl_3_, 10 mol % of DPPF, 3.0 equiv of Cs_2_CO_3_, toluene, reflux) led to (4-imino-3,4-dihydroquinazolin-2-yl)phosphonates **203** in 37–78% yields (Scheme 42). This process involves an initial nucleophilic addition of phosphite to carbodiimide **197** affording intermediate **200** which undergoes an oxidative addition of Pd(0) to give Pd(II) species **201**. Subsequently, the insertion of isocyanide **199** to species **201** affords intermediate **202**, which finally generates phosphorylated quinazoline **203** through reductive elimination.

**Scheme 43:**
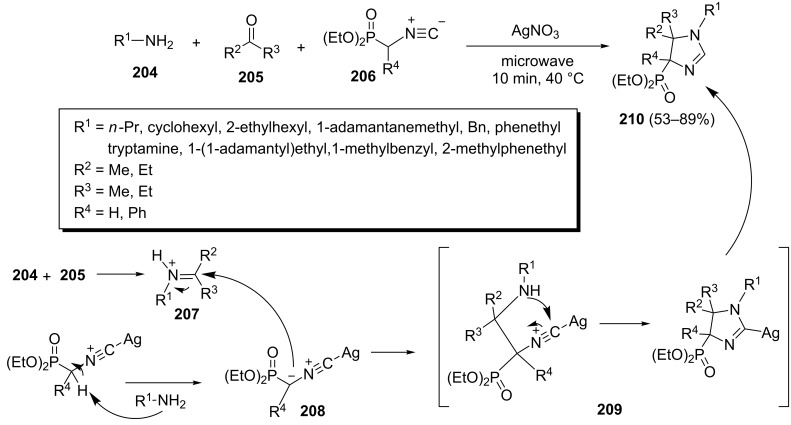
Silver-catalyzed three-component synthesis of (2-imidazolin-4-yl)phosphonates.

A silver-catalyzed three-component reaction of α-isocyanophosphonates **206**, ketones **205** and amines **204** under microwave irradiation to afford (2-imidazolin-4-yl)phosphonates **210** has recently been reported (Scheme 43) [81]. The yields of the products under optimized conditions were 53–89%. This process involved a Mannich-type addition of a silver-activated α-isocyanophosphonate anion **208** to an iminium salt **207**, resulting from reaction of amine **204** and ketone **205**, to give intermediate **209** which cyclizes to afford (2-imidazolin-4-yl)phosphonates **210**.

### 6 1,3-Dipolar cycloaddition-based multicomponent reactions

1,3-Dipolar cycloaddition-based multicomponent reactions usually involve the cycloaddition of in situ generated 1,3-dipoles and dipolarophiles to give five-membered heterocycles. In recent years, many heterocyclic phosphonates have been prepared via this efficient method.

**Scheme 44:**
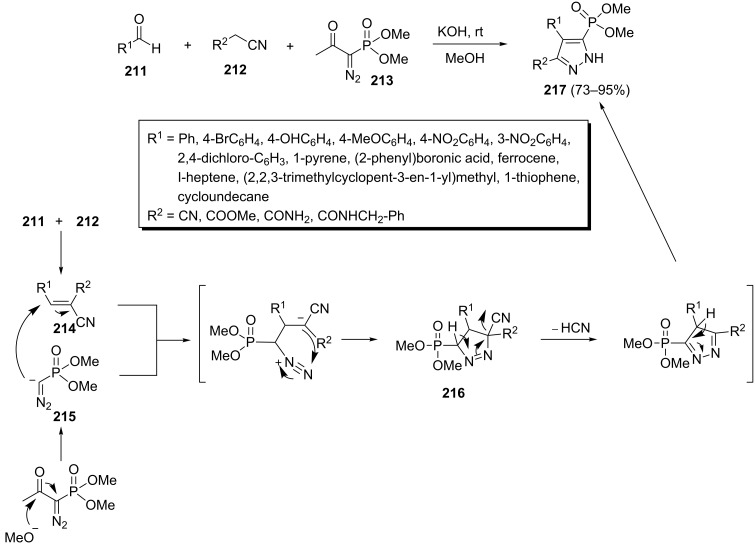
Three-component synthesis of phosphonylpyrazoles.

One of the best strategies is based on the use of Bestmann–Ohira reagent (BOR) as the 1,3-dipole precursor. Smietana et al. accomplished a related three-component reaction with different aldehydes **211**, nitrile derivatives **212** and dimethyldiazomethylphosphonate **213** to prepare phosponylpyrazoles **217** in the presence of KOH in MeOH in 73–95% yields (Scheme 44) [82]. Based on their explanation, the treatment of dimethyldiazomethylphosphonate **213** with a nucleophilic base generates diazo compound **215**. The subsequent [3 + 2] cycloaddition reaction of **215** with Knoevenagel adduct **214**, resulting from condensation of aldehydes **211** and nitrile derivatives **212**, lead to cyclo-adduct intermediates **216** which cyclize to phosphorylated pyrazoles **217**.

**Scheme 45:**
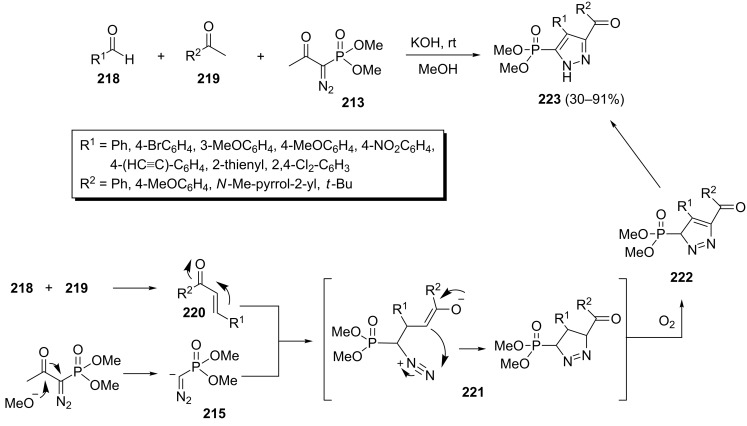
One-pot three-component synthesis of 3-carbo-5-phosphonylpyrazoles.

In this way, a one-pot three-component reaction of aldehydes **218**, methyl ketones **219** and the Bestmann–Ohira reagent has been developed for the preparation of different 3-carbo-5-phosphonylpyrazoles **223** (Scheme 45) [83]. The corresponding phosphonylpyrazoles **223** were formed via a Claisen–Schmidt/1,3-dipolar cycloaddition/oxidation process under basic conditions in MeOH in 30–91% yields.

**Scheme 46:**
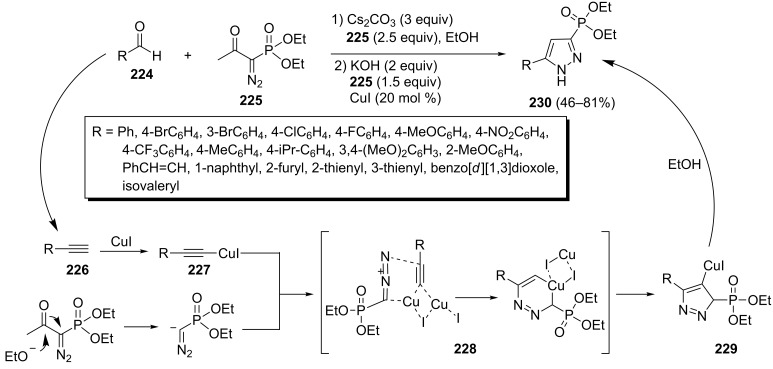
A one-pot two-step method for the synthesis of phosphonylpyrazoles.

The dual reactivity of diethyl (1-diazo-2-oxopropyl)phosphonate (**225**) in a one-pot, two-step three-component method for the synthesis of phosphonylpyrazoles has been presented by Kumar et al. The phosphonate **225** acted both as a 1,3-dipole precursor and as a Horner–Wadsworth–Emmons (HWE) reagent. Therefore, the reaction of phosphonate **225** with aldehydes **224** generated terminal acetylenes **226** which cyclized with the second molecule of phosphonate **225** in the presence of Cu(I) to afford (5-methyl-1*H*-pyrazol-3-yl)phosphonates **230** in 46–81% yields (Scheme 46) [84].

**Scheme 47:**
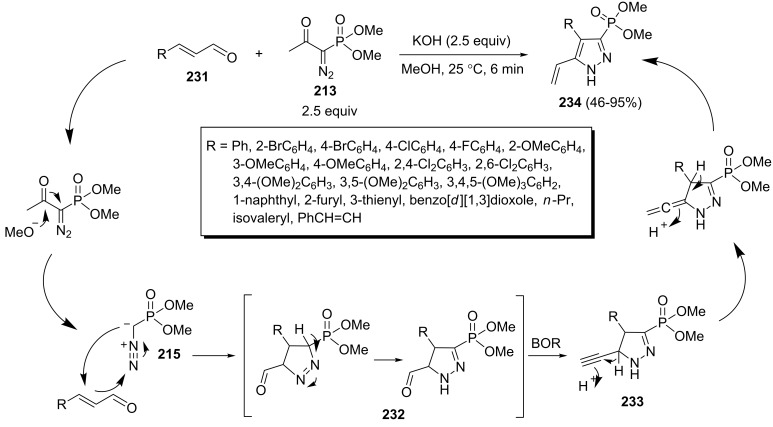
A one-pot method for the synthesis of (5-vinylpyrazolyl)phosphonates.

Also, a domino reaction based on the dual reactivity of BOR as a homologation reagent as well as cycloaddition reactant for the synthesis of (5-vinylpyrazolyl)phosphonates **234** from α,β-unsaturated aldehydes **231** has been reported (Scheme 47) [85]. The desired vinylpyrazoles **234** were obtained in 46–95% yields under optimized conditions (2.5 equiv BOR, 2.5 equiv KOH, 25 °C, 6 min, MeOH). In this reaction, the generated diazomethyl anion **215** underwent a 1,3-dipolar cycloaddition with α,β-unsaturated aldehydes **231** to give pyrazolinecarboxaldehyde **232**. The subsequent reaction of aldehyde **232** with another molecule of BOR afforded pyrazoline alkyne intermediate **233** which, after a 1,3-hydrogen shift, aromatized to vinylpyrazoles **234**.

**Scheme 48:**
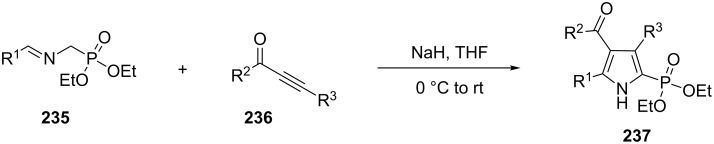
Synthesis of 1*H*-pyrrol-2-ylphosphonates via the [3 + 2] cycloaddition of phosphonate azomethine ylides with ynones.

Recently, the [3 + 2] cycloaddition of phosphonate azomethine ylides **235** with ynones **236** to give substituted 1*H*-pyrrol-2-ylphosphonates **237** has been described by Yu et al. (Scheme 48) [86].

**Scheme 49:**
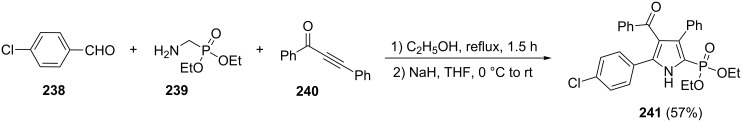
Three-component synthesis of 1*H*-pyrrol-2-ylphosphonates.

The desired 1*H*-pyrrol-2-ylphosphonate **241** could also be obtained through the three-component reaction of 4-chlorobenzaldehyde (**238**), aminomethylphosphonate **239** and ynones **240** in 57% yield (Scheme 49).

### 7 Reissert-type multicomponent reactions

**Scheme 50:**
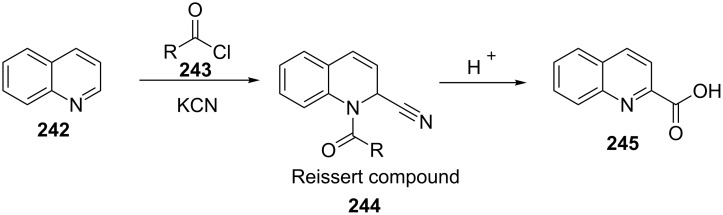
The classical Reissert reaction.

The traditional Reissert reaction is a one-pot treatment of quinoline (**242**) with acid chlorides **243** and KCN to afford Reissert compound **244** which can be hydrolyzed to give quinoline-2-carboxylic acid (**245**) (Scheme 50) [87].

**Scheme 51:**
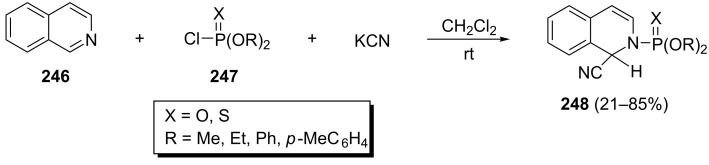
One-pot three-component synthesis of *N*-phosphorylated isoquinolines.

This reaction can also be applied to isoquinolines and some pyridines. Additionally, a wide range of activating groups such as chloroformates, acetylenic esters, R_3_SiOTf, Tf_2_O and various nucleophiles can be utilized in this reaction. For example, the one-pot reaction of isoquinoline (**246**) with KCN and chlorophosphates or chlorothiophosphates **247** has been described by Spatz and Popp. The corresponding *N*-phosphorylated isoquinolines **248** were obtained in 21–85% yields in CH_2_Cl_2_ at room temperature (Scheme 51) [88].

**Scheme 52:**
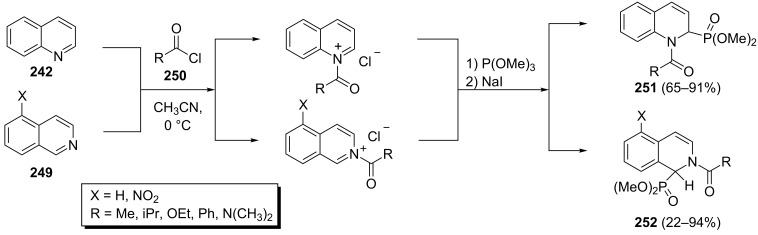
One-pot three-component synthesis of 1-acyl-1,2-dihydroquinoline-2-phosphonates and 2-acyl-1,2-dihydroisoquinoline-1-phosphonates.

Further, 1-acyl-1,2-dihydroquinoline-2-phosphonates **251** and 2-acyl-1,2-dihydroisoquinoline-1-phosphonates **252** have been prepared via the one-pot reaction of quinoline (**242**) or isoquinolines **249** with acyl chlorides **250** and trimethyl phosphite in the presence of NaI in 22–94% yields depending on the nature of the acyl chlorides and substituents X (Scheme 52) [89–90]. The three-component reaction of dimethyl phosphite, acetyl chloride and isoquinoline under refluxing in CH_2_Cl_2_ in the presence or absence of triethylamine led to the desired 1,2-dihydroisoquinoline-1-phosphonates in 66% and 45% yields, respectively. However, the reaction of trimethyl phosphite, acetyl chloride and isoquinoline in MeCN at 0 °C followed by heating at 50 °C gave the corresponding 1,2-dihydroisoquinoline-1-phosphonate in 85% yield.

**Scheme 53:**
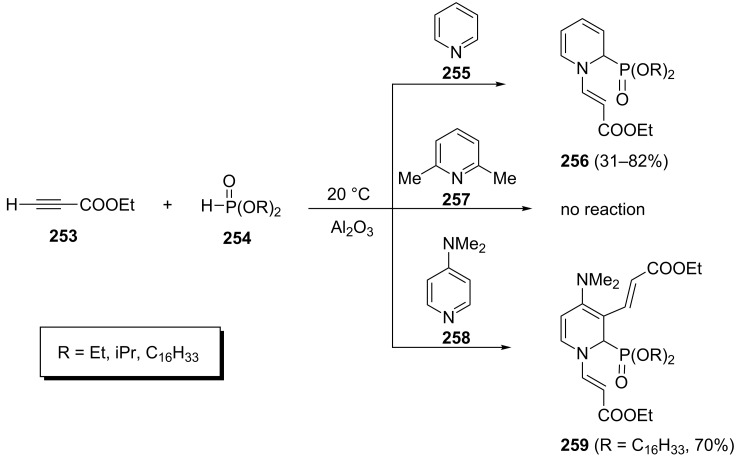
Three-component reaction of pyridine derivatives with ethyl propiolate and dialkyl phosphonates.

Albouy et al. discovered an unexpected route towards 1,2-dihydropyridinylphosphonates during their studies of the base-catalyzed Pudovik reaction [91]. Unlike most other bases, the pyridine-mediated reaction of ethyl propiolate (**253**) and dialkyl phosphonates **254** led to 1,2-dihydropyridinylphosphonates **256**. Thus, the 1,2-dihydropyridinylphosphonates **256** were synthesized via a one-pot three-component reaction of pyridine **255**, ethyl propiolate (**253**) and dialkyl phosphonates **254** in moderate to good yields at 20 °C (Scheme 53). Under similar conditions, no reaction occurred with 2,6-lutidine (**257**) whereas 4-dimethylaminopyridine (**258**, DMAP) was efficiently converted to dialkylated 1,2-dihydropyridine-3-phosphonate **259**.

**Scheme 54:**
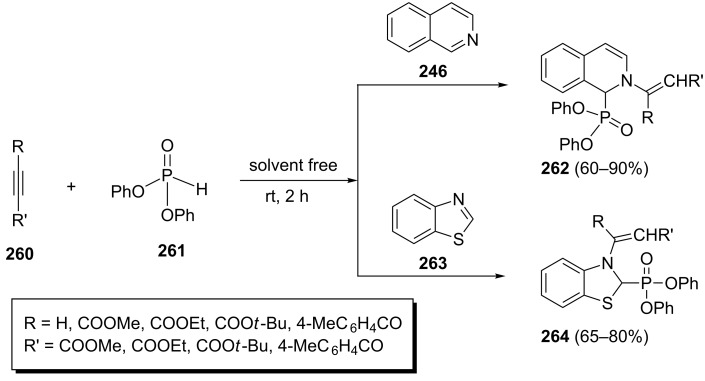
Three-component reactions for the phosphorylation of benzothiazole and isoquinoline.

Yavari et al. have described the phosphorylation of benzothiazole (**263**) and isoquinoline (**246**) through a one-pot three-component reaction with activated acetylenes **260** and diphenyl phosphonate (**261**) under solvent-free conditions at room temperature (Scheme 54) [92]. Moderate to good yields (60–90%) of the desired heterocyclic phosphonates **262** and **264** were obtained.

**Scheme 55:**
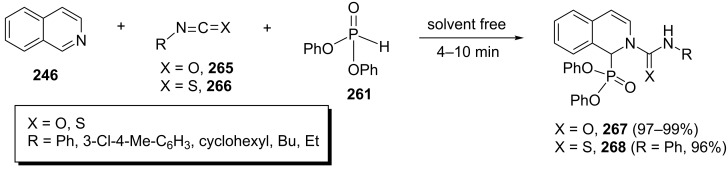
Three-component synthesis of diphenyl [2-(aminocarbonyl)- or [2-(aminothioxomethyl)-1,2-dihydroisoquinolin-1-yl]phosphonates.

The same group has also reported another Reissert-type reaction for the synthesis of 1,2-dihydroisoquinolin-1-ylphosphonates via activation of isoquinoline with isocyanate or isothiocyanate. In this case, the one-pot three-component reaction of isoquinoline and diphenyl phosphite with isocyanates **265** or isothiocyanates **266** furnished (dihydroisoquinolin-1-yl)phosphonates **267** or **268** in 96–99% yields under solvent-free conditions at room temperature (Scheme 55) [93].

**Scheme 56:**
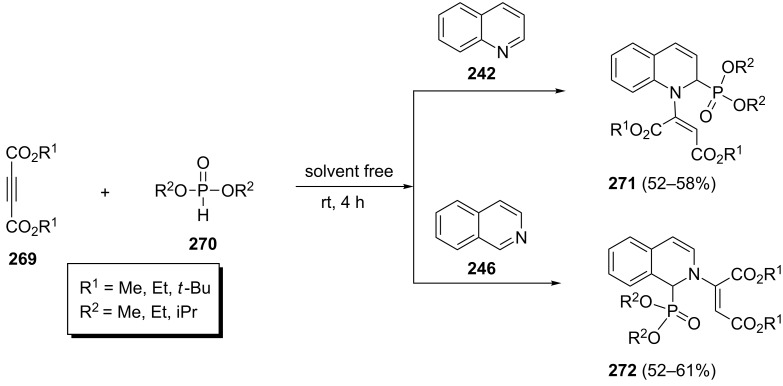
Three-component stereoselective synthesis of 1,2-dihydroquinolin-2-ylphosphonates and 1,2-dihydroisoquinolin-1-ylphosphonates.

A solvent-free stereoselective synthesis of 1,2-dihydroquinolin-2-ylphosphonates **271** and 1,2-dihydroisoquinolin-1-ylphosphonates **272** via the three-component reactions of quinoline or isoquinoline, dialkyl acetylenedicarboxylates **269**, and dialkyl phosphonates **270** has been described by Shaabani et al. (Scheme 56) [94]. The corresponding products **271** and **272** were isolated in 52–61% yields and their nOe analysis revealed the geometry of the alkene bonds to be E .

**Scheme 57:**
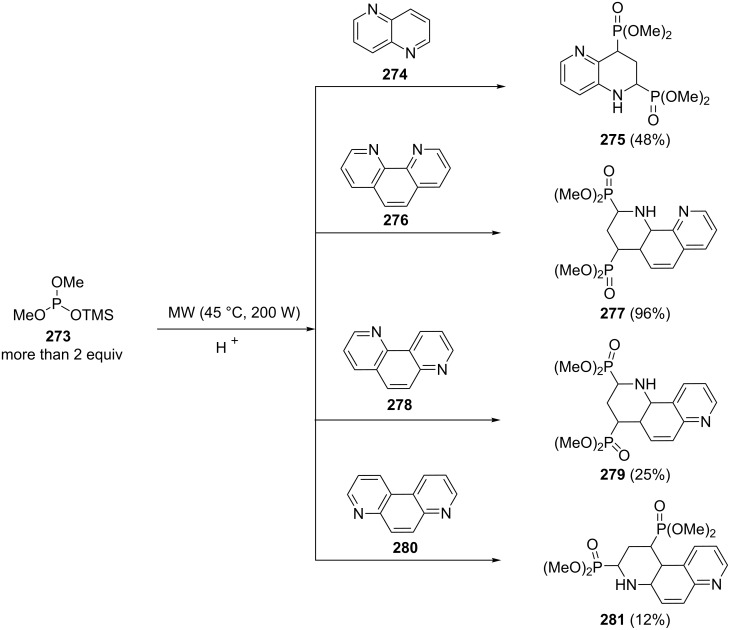
Diphosphorylation of diazaheterocyclic compounds via a tandem 1,4–1,2 addition of dimethyl trimethylsilyl phosphite.

A tandem 1,4–1,2 addition of dimethyl trimethylsilyl phosphite (DMPTMS, **273**) to diazaheterocyclic compounds under microwave irradiation in acidic medium led to diphosphorylated products [95]. The 1,5-naphthyridine **274** and phenanthrolines **276**, **278** and **280** in the presence of more than 2 equiv of DMPTMS were converted to the corresponding diphosphorylated products **275**, **277**, **279** and **281** with a high diastereoisomeric ratio (Scheme 57). In this reaction, the 1,4-addition of DMPTMS as a nucleophilic reagent on the N-protonated heterocycle followed by a 1,2-addition of DMPTMS on the N-silylated species lead to the diphosphorylated heterocycles after aqueous work-up.

### 8 Miscellaneous multicomponent reactions

**Scheme 58:**
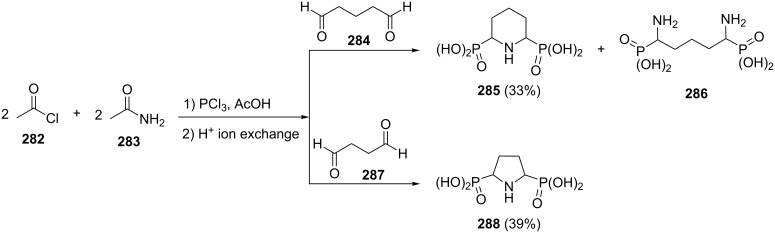
Multicomponent reaction of alkanedials, acetamide and acetyl chloride in the presence of PCl_3_ and acetic acid.

The reaction of pentanedial (**284**) with acetamide (**283**) and acetyl chloride (**282**) in the presence of PCl_3_ and acetic acid gives a 1:1 mixture of piperidinyldiphosphonic acid **285** and acyclic (diaminoalkyl)diphosphonic acid **286**. However, butanedial (**287**) under similar conditions affords exclusively pyrrolidinyldiphosphonic acid **288** in 39% yield (Scheme 58) [96].

**Scheme 59:**
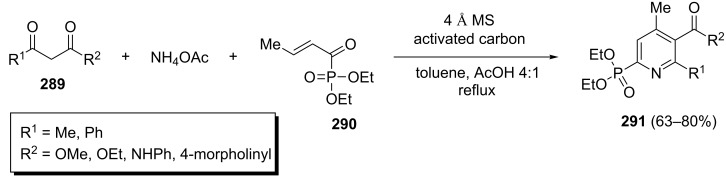
An oxidative domino three-component synthesis of polyfunctionalized pyridines.

An oxidative domino three-component reaction of α-ketophosphonates **290**, ammonium acetate and various 1,3-dicarbonyl compounds **289** to give pyridinylphosphonates **291** has been described. This method allowed the synthesis of highly functionalized pyridinylphosphonates **291** in 63–80% yields in refluxing AcOH/toluene 4:1 in the presence of 4 Å molecular sieves (Scheme 59) [97].

**Scheme 60:**
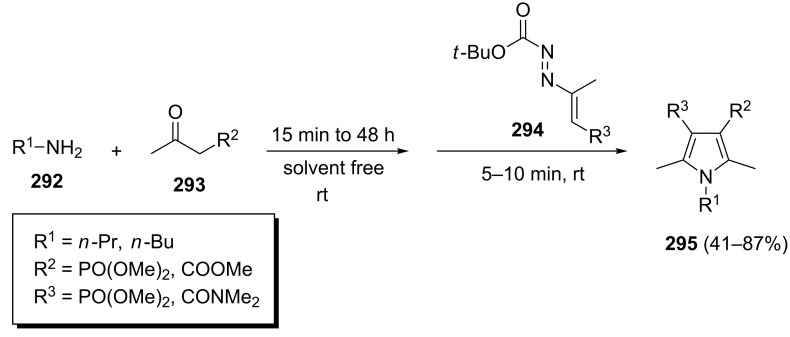
A sequential one-pot three-component synthesis of polysubstituted pyrroles.

A sequential three-component enamine–azoene annulation reaction of primary aliphatic amines **292**, activated methylene compounds **293**, and 1,2-diaza-1,3-dienes (DDs, **294**) has been reported to give polysubstituted pyrroles **295** (Scheme 60) [98]. The desired phosphono-substituted pyrroles were isolated in 41–87% yield under solvent and catalyst-free conditions.

**Scheme 61:**
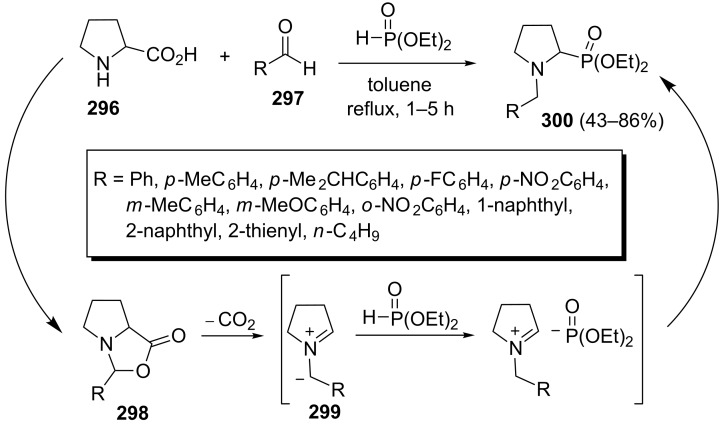
Three-component decarboxylative coupling of proline with aldehydes and dialkyl phosphites for the synthesis of pyrrolidinylphosphonates.

Kaboudin et al. described a three-component, catalyst-free decarboxylative coupling of proline (**296**) with aldehydes **297** and dialkyl phosphonates to afford pyrrolidinylphosphonates **300**. The corresponding pyrrolidinylphosphonates **300** were isolated in 43–86% yields under refluxing in toluene (Scheme 61) [99]. The reaction was proposed to proceed through the condensation of proline with an aldehyde under formation of oxazolidin-5-ones **298** followed by decarboxylation to give the azomethine ylides **299**. Finally, the addition of dialkyl phosphonate to the azomethine ylides **299** afforded pyrrolidinylphosphonates **300**.

**Scheme 62:**
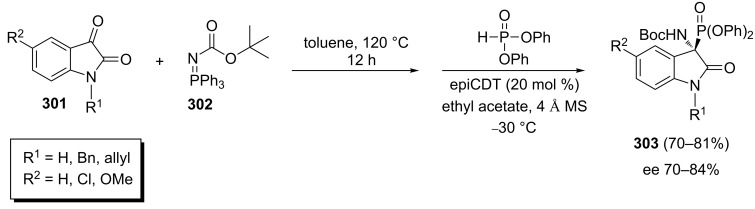
Three-component domino aza-Wittig/phospha-Mannich sequence for the phosphorylation of isatin derivatives.

An efficient protocol comprising a domino aza-Wittig/phospha-Mannich sequence for the phosphorylation of isatin derivatives has been reported by Kumar et al. According to this method, the one-pot reaction of isatin derivatives **301**, iminophosphorane **302**, and diphenyl phosphonate in the presence of Cinchona-derived thiourea as the catalyst afforded α-aminophosphonates **303** in 70–81% yields and with 70–84% ee (Scheme 62) [100].

**Scheme 63:**
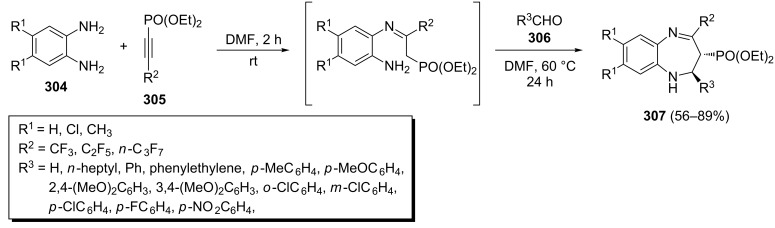
Stereoselective synthesis of phosphorylated *trans*-1,5-benzodiazepines via a one-pot three-component reaction.

The *trans*-1,5-benzodiazepines **307** bearing both, perfluoroalkyl and phosphonate groups, were stereoselectively synthesized through a one-pot three-component condensation of *o*-phenylenediamines **304**, fluorinated alkynylphosphonates **305** and aldehydes **306** (Scheme 63) [101]. The corresponding 1,5-benzodiazepines **307** were isolated in 56–89% yields under optimized conditions. In this reaction aromatic aldehydes afforded slightly higher yields than aliphatic aldehydes. Also, the yields of aromatic aldehydes bearing electron-donating substituents were higher than those bearing electron-withdrawing substituents.

**Scheme 64:**
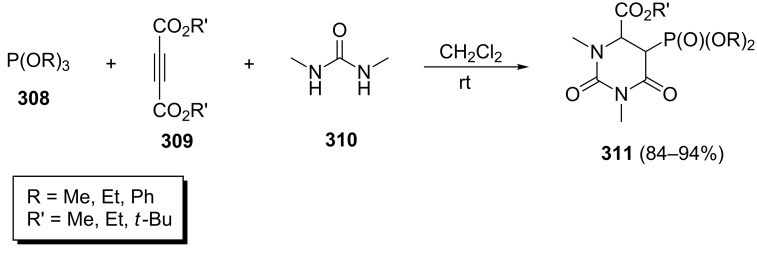
One-pot three-component synthesis of phosphorylated 2,6-dioxohexahydropyrimidines.

Yavari et al. described the synthesis of phosphorylated 2,6-dioxohexahydropyrimidines **311** via a three-component reaction [102]. This method involved the one-pot reaction of *N*,*N'*-dimethylurea (**310**) and dialkyl acetylenedicarboxylates **309** in the presence of trialkyl phosphites **308** at room temperature (Scheme 64). The desired products were obtained in high yields between 84 and 94%.

## Conclusion

In this article the use of different multicomponent reactions (MCRs) for the synthesis of heterocyclic phosphonates has been reviewed. This review demonstrates the synthetic potential of multicomponent reactions for the construction of phosphono-substituted heterocyclic rings. The Kabachnik–Fields reaction can be considered the starting point of multicomponent synthesis of this class of compounds. However, the major advancements in this interesting field have been achieved in recent years. More than 75% of the cited literature in this review has been published within the last six years, of which more than three quarters dealt with the synthesis of new heterocyclic phosphonates from non-heterocyclic phosphorus reagents. The remaining works reported the phosphorylation of parent heterocyclic systems. It is worth mentioning, that most of the cited publications focused on the synthesis of five and six-membered rings and only four articles described the synthesis of three and seven-membered heterocycles. Additionally, the majority of the reported syntheses were devoted to the development of new methodologies including the use of advanced catalytic systems, alternative solvents and microwave irradiation. Thus, the development of novel MCR based on phosphorous reagents would allow the synthesis of macrocyclic and medium or large-sized heterocyclic systems, substances which are currently underrepresented in the literature. Further, the design of new biocompatible scaffolds such as β-lactams and peptidomimetics possessing phosphonate groups by MCR-based strategies would significantly extend the synthetic potential of MCRs towards heterocyclic phosphonates.
